# Feasibility of a Lateral Flow Test for Neurocysticercosis Using Novel Up-Converting Nanomaterials and a Lightweight Strip Analyzer

**DOI:** 10.1371/journal.pntd.0002944

**Published:** 2014-07-03

**Authors:** Paul L. A. M. Corstjens, Claudia J. de Dood, Jeffrey W. Priest, Hans J. Tanke, Sukwan Handali, and the Cysticercosis Working Group in Peru

**Affiliations:** 1 Department of Molecular Cell Biology, Leiden University Medical Center, Leiden, The Netherlands; 2 Division of Parasitic Diseases and Malaria, Centers for Disease Control and Prevention, Atlanta, Georgia, United States of America; Institute of Tropical Medicine of Antwerp, Belgium

## Abstract

Neurocysticercosis is a frequent parasitic infection of the human brain, occurring in most of the world, and requires imaging of the brain to diagnose. To determine the burden of disease and to simplify diagnosis, a field-friendly rapid lateral flow (LF) based antibody screening test was developed. The assay utilizes novel nano-sized up-converting phosphor (UCP) reporter particles in combination with a portable lightweight analyzer and detects antibodies in serum samples reactive with bacterial-expressed recombinant (r) T24H, a marker for detecting neurocysticercosis cases. Three sequential flow steps allow enrichment of antibodies on the Test (T) line and consecutive binding of protein-A coated UCP reporter particles. Antibody binding was determined by measuring 550 nm emission after excitation of the UCP label with a 980 nm infrared (IR) diode. Clinical sensitivity and specificity of the assay to detect cases of human neurocysticercosis with 2 or more viable brain cysts were 96% and 98%, respectively, using a sample set comprised of sera from 63 confirmed cases and 170 healthy parasite-naïve non-endemic controls. *In conclusion*: Proof-of-principle, of a rapid UCP-LF screening assay for neurocysticercosis was demonstrated. The assay utilized bacterial-expressed rT24H as a potential alternative for baculovirus-expressed rT24H. Performance of the UCP-LF assay was excellent, although further studies need to confirm that bacterial expressed antigen can entirely replace previously used baculovirus antigen. In addition, the increasing availability of commercial sources for UCP reporter materials as well as the accessibility of affordable semi-handheld scanners may allow UCP-based bioanalytical systems for point-of-care to evolve at an even faster pace.

## Author Summary

A field-friendly screening tool to simplify serological diagnosis of neurocysticercosis was developed. The assay utilizes a rapid lateral flow based test platform in combination with unique fluorescent label, up-converting phosphor (UCP) reporter particles. The UCP reporter technology uses infrared excitation and emission of higher energy visible light. Total absence of autofluorescence provides the label with a distinctive advantage compared to common fluorescent labels. The developed assay detects antibodies against an established marker for detecting neurocysticercosis cases, recombinant T24H. In this study rT24H was produced by a bacterial expression system, a less cumbersome method compared to the previously used baculovirus expression system. With a sample set comprised of 63 confirmed neurocysticercosis cases and 170 healthy controls, clinical sensitivity and specificity were 96% and 98%, respectively. The study shows proof-of-principle that the designed UCP-rT24H assay can provide an on-site rapid screening method utilizing a mobile lightweight scanner and 40 nm yttrium fluoride UCP particles. A semi-handheld UCP reader and nano-sized UCP particles performed as good as previously used ‘research and development’-only tools. Availability of commercial sources for UCP reporter materials as well as the accessibility to affordable scanners may allow UCP-based bioanalytical systems for point-of-care to evolve at an even faster pace.

## Introduction

Cysticercosis is a tissue infection caused by hatched oncospheres, a larval form of the pork tapeworm *Taenia solium*. Although caused by the same pathogen, cysticercosis is different from the carriage of the adult tapeworm in the intestine (taeniasis). Cysticercosis is mainly a food borne disease occurring after exposure to *T. solium* eggs. Upon ingestion, the eggs release oncospheres that are able to invade the intestinal wall and circulate through the bloodstream. This can result in neurocysticercosis, invasion of the nervous system and the formation of cysts in the brain, which is a major cause of adult acquired epilepsy and other neurological morbidity in many areas of the world [1], [2]. Diagnostic imaging of the central nervous system is required to confirm the diagnosis and the type of disease [3]. The availability of a rapid serological diagnosis that targets stage-specific antibodies for human cysticercosis is considered very helpful in control programs for estimating the burden (sero-prevalence) of disease in susceptible population groups. A low-cost rapid diagnostic test could also be applied to determine seroprevalence rates in pigs to assess interruption of transmission.

Currently, the most useful and the best documented serological test is the enzyme immunoelectrotransfer blot (EITB), which relies on antibody reactivity with 7 diagnostic lentil lectin purified glycoproteins (LLGP) [4]. Recombinant or synthetic peptide molecules of these proteins are available [5]–[7]; rT24H corresponding to a 24,000 Da protein of the LLGP extract [7] was identified as the recombinant protein providing the best sensitivity and specificity for detecting neurocysticercosis using a multi-antigen print immunoassay (MAPIA) [8]. Although the EITB test is the reference standard it is not widely available nor does it exist in point-of-care or field-friendly formats. In this study we have developed a LF-based serological test that can be used for point-of-care (POC) applications and settings with minimal infrastructure [9], [10]. In comparison to an earlier developed immunochromatography assay based on superparamagnetic particles [11], the assay described here is fully portable including a lightweight LF strip analyzer, and is suitable for worldwide shipping and storage at ambient temperature [12].

Detection devices available for advanced testing, use scanners to measure reflectance, contrast, color change, or fluorescence [13], [14] and improve clinical sensitivity. Superior sensitivity may also permit better detection of antibodies in other biological matrices, such as saliva, which can be obtained non-invasively [15], [16]. In this respect the potential of up-converting reporter (UCP) materials for POC diagnostics has been recognized [17], [18] and several studies have demonstrated improved sensitivity and robustness of the technology [19]–[23], including use in LF-based assays [24]–[29]. Much of the improvement is related to the unique features of the UCP reporter label, which include emission of higher energy visual light upon excitation with lower energy 980 nm infrared light [30] by a process called up-conversion. In contrast to conventional fluorescent labels, applications with UCP are not hampered from background fluorescence (auto-fluorescence). Moreover, the UCP label does not fade, allowing LF strips to be stored as a permanent record. In addition, interference from hemolysis of red blood cells is not seen with the UCP detection system, a problem sometimes encountered in rapid assays using finger stick blood.

The UCP-LF assay described here detects human antibodies against the rT24H antigen. This antigen is similar to previously reported baculovirus produced rT24H antigens [7] but in this study rT24H produced in a bacterial expression system provides a more convenient production method. For the current study we have adapted an earlier described UCP-based antibody test [31], [32] for applications with up-converting nano material ([33]–[36]) and a portable lightweight LF strip scanner platform with an integrated infrared (IR) diode to analyze UCP-LF strips. Results were compared to previously used reference materials and bench top readers. The described assay format is suited for POC and on-site testing applications and integration in microfluidic devices [37]–[39].

## Materials and Methods

### Ethics statement

All clinical samples used in this study were collected in previous studies with specific permission for future use of stored samples. Samples were anonymized and the study was performed in compliance with protocols approved by the ethical review boards of all participating institutes. The defined cysticercosis serum samples were obtained at the Instituto de Ciencias Neurologicas (Lima, Peru) and samples from healthy controls were obtained from Dutch Blood bank donors (Leiden, The Netherlands) and U.S. residents (Atlanta, Georgia).

### Serum samples and patient population

#### Sera for assay optimization

A series of standards, 0, 1, 2.5 10 and 100 arbitrary units per µL (Units) was constructed from a pool of 5 serum samples from human cases with confirmed cysticercosis by dilution in a negative serum pool (combined serum samples from United States residents with no history of international travel). The samples contained antibodies that reacted to the diagnostic proteins in the LLGP-EITB assay [4], with the 2.5 Units sample representing the lower limit of detection level. Normal human serum (NHS) was obtained from Innovative Research (Dunn Labortechnik, Asbach Germany).

#### Serum panels to determine clinical sensitivity and specificity

A total of 63 banked serum samples from patients with confirmed neurocysticercosis were used to validate the assay. Samples were previously collected by the Cysticercosis Working Group (CWG) in Peru Lima. The definitive diagnosis of neurocysticercosis was confirmed by CT or MRI brain imaging [40] and for this study, we chose samples from patients with 2 or more viable cysts. A total of 170 serum samples collected in regions where transmission of cysticercosis does not occur were used to assess specificity. These samples consisted of a panel of 78 serum samples assembled from healthy residents of the United States and a panel of 92 serum samples from blood bank donors in the Netherlands.

#### Serum samples with heterologous infections

A set of 80 samples from previous studies [8], [11] was tested to evaluate potential cross reactivity with other worm infections.

#### Serum samples used to compare baculovirus and bacterial rT24H antigen

A set of 39 banked serum samples from previous studies [8], [11] was used for a direct comparison of the baculovirus- and bacterial-expressed antigens in the ELISA format (see additional Supporting Information).

#### Dilution of the serum samples

For UCP-LF assays, the frozen serum samples were thawed and centrifuged for 2 min at 14,000 rpm. The supernatants were the diluted to 2.5% v/v in LF assay buffer (100 mM HEPES pH 7.2, 270 mM NaCl, 0.5% w/v Tween-20, 1% w/v bovine serum albumin) and 40 µL of this was applied to the LF strips (see description of the UCP-rT24H assay).

### Expression and purification of recombinant rT24H

The hydrophilic extracellular domain of T24, T24H [6], was PCR amplified using AmpliTaq Gold DNA polymerase (Perkin-Elmer Cetus, Foster City, CA) using the following forward and reverse deoxyoligonucleotide primers: 5′-CGC AGA TCT TAT CGT CAC GAT TTC GTT CGC C-3′ and 5′-GCG GAA TTC CGC CGA AGG CCA GAG CGG AAT CCT TC-3′, respectively. In these sequences, the restriction sites used for cloning (*Bgl* II and *EcoR* I, respectively) are underlined. The PCR amplification protocol and the techniques for directional cloning into the *BamH* I and *EcoR* I sites of a modified pGEX 4T-2 vector have previously been described [41]. Expression in the modified vector yielded a recombinant protein with glutathione-S-transferase (GST) and a thrombin cleavage site on the amino terminus and a 6× polyhistidine tag and factor Xa cleavage site on the carboxy terminus. The plasmid was transformed into *Escherichia coli* HB101 cells (Promega Corp., Madison, WI), and sequenced with both forward and reverse primers to confirm sequence fidelity and orientation. Expression of the His tagged protein was performed in *Escherichia coli* strain BL21(DE3)pLysS (Agilent Technologies, LaJolla, CA), grown in LB broth at 37°C, shaking at 225 rpm. After reaching a culture density of OD_600 nm_∼0.5–0.6, IPTG was added at a final concentration of 0.2 mM and incubation was continued for 3 h. The cells were collected by centrifugation (5K, 4°C for 20 min) and the cell pellet was stored at −80°C. Recombinant protein was purified on a 10 mL GST–Sepharose 4B affinity column as directed by the manufacturer (GE Healthcare, Piscataway, NJ), and glutathione-eluted material was dialyzed against PBS (Spectra/Por3; 3,500-Da cutoff; Spectrum Laboratories, Rancho Dominguez, CA). His-tagged protein was further purified over a 5 ml Ni^2+^ affinity column (GE Healthcare) equilibrated in PBS and elution with a step gradient of 250 mM imidazole in PBS. After a second dialysis step the purified His-tagged protein was treated with bovine Factor Xa (approximately 1 U/mg protein; Novagen, San Diego, CA) overnight at room temperature to remove the His-tag; the cleaved His-tag and uncleaved proteins were then removed by reapplication onto Ni^2+^ columns. GST-tagged rT24H (without His tag) was eluted with 2 mM imidazole in PBS at pH 7.2. The purified protein was dialyzed a third time against PBS prior to storage at −80°C (see Supporting Information for gel analysis of the purified GST-tagged protein used to prepare the rT24H Test line of the LF strips).

### UCP reporter and LF strip materials

**Figure 1:**
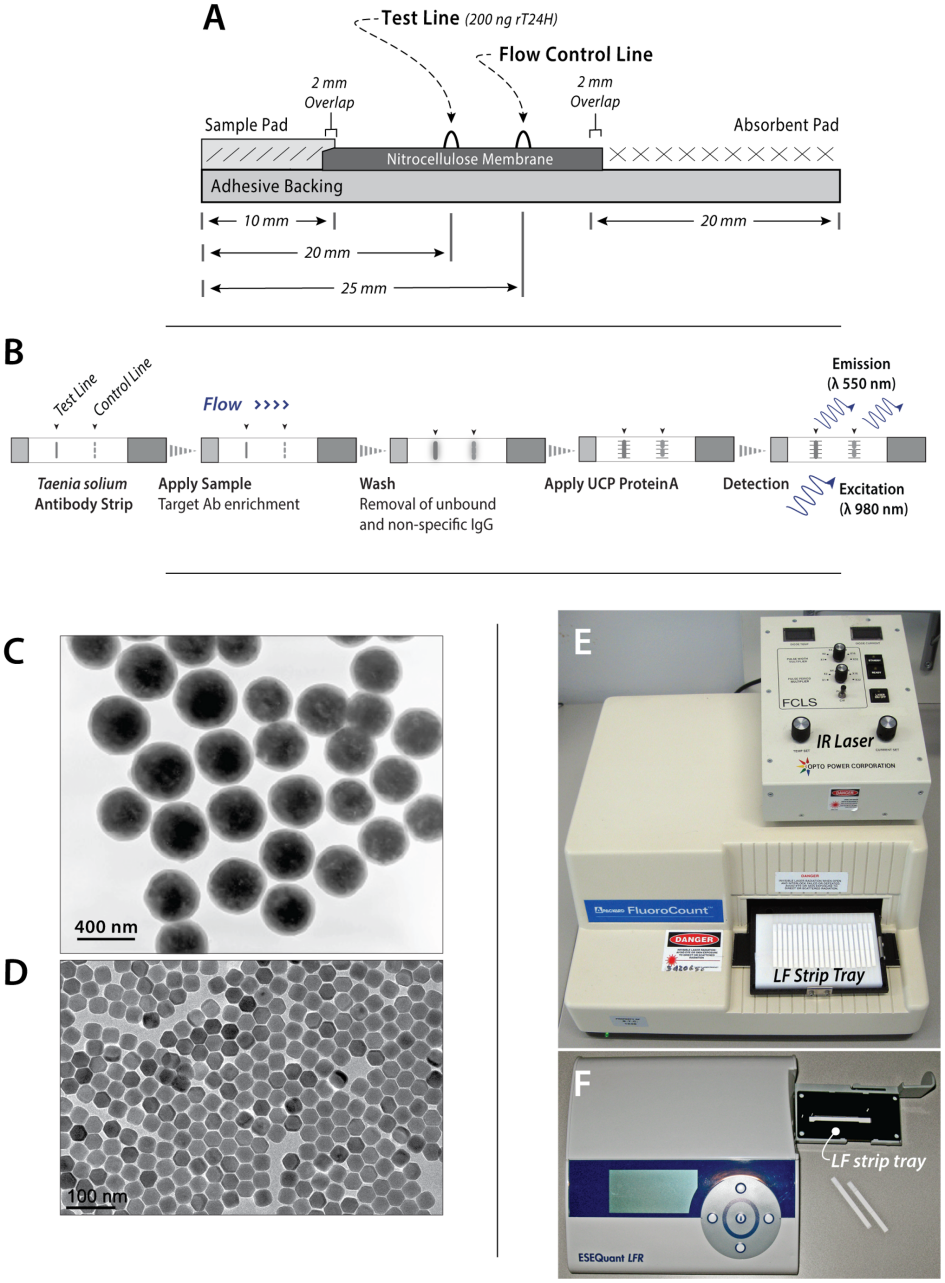
The UCP-rT24H assay. Panel A: Schematic of the UCP-rT24H LF strip: Test line (T) 200 ng rT24H and Flow Control line (FC) 100 ng protein-A. Panel B: The LF protocol for antibody detection (in previous publications referred to as consecutive flow, CF [31] with the three sequential flow steps indicated. Panel C and D: Transmission electron microscopy images of respectively, the 400 nm [44] and 40 nm (by courtesy of J. Collins) UCP materials. Panel E and F: Image of respectively, the modified Fluorocount Packard benchtop reader for scanning multiple LF strips and the portable, custom adapted, lightweight ESEQuant *LFR* reader.

EDC (1-ethyl-3-(3-dimethylaminopropyl) carbodiimide HCL) and sulfo-NHS (*N*-hydroxy-sulfosuccinimide) were purchased from Pierce (Thermo Scientific, Rockford, IL). Protein A (ProtA; recombinant RPA-50; Repligen, Waltham, MA, USA) was covalently bound to UCP reporters as described earlier [25], [42] in a ratio of 25 µg/mg for the submicron-sized silica coated and C10-carboxylated 400 nm Y_2_O_2_S:Yb^3+^,Tm^3+^ particles (OraSure Technologies, Bethlehem, PA, USA; [27], [43]–[45] and 250 µg/mg for poly(acrylic acid) coated the nano-sized 40 nm NaYF_4_:Yb^3+^,Er^3+^ UCP particles (Intelligent Materials Solution, Princeton, NJ, USA). Bovine serum albumin was obtained from Sigma Aldrich (A-2153). The resulting UCP-ProtA conjugates (1 mg/mL) were stored in a refrigerator at 4°C and used over a period of 6 months without notable loss of performance. LF strip materials were obtained from Merck-Millipore (25 mm, laminated Hi-Flow Plus HF090 nitrocellulose and 1 cm Surewick glass-fiber sample pad) and GE Healthcare (Whatman #470 absorbent pad). Materials were compiled on an adhesive plastic backing card (G&L Precision Die Cutting, San Jose, CA, USA). LF cards were prepared with a Test (T) line of 200 ng (per 4 mm width) rT24H and a Flow Control (FC) line of100 ng (per 4 mm width) protein-A according previously described protocols [25], [42] and cut into 4 mm wide LF strips using a BioDot CM4000 guillotine cutter. A schematic of the LF strip is shown in Fig. 1. Strips were stored dry in containers with silica.

### rT24H ELISA

Microwell plates (Immulon 2 HB) were sensitized with rT24H antigen (0.6 µg/mL in Tris buffer (0.05 M Tris/HCl pH 8.0, 0.3 M KCl, 2 mM EDTA) for 2 hours at room temperature on a plate shaker. Following antigen sensitization, the plates were washed 4 times with PBS with 0.3% v/v Tween-20 (PBS-Tween). Plates were blocked for 30 min with 100 µL of StabilCoat® Immunoassay Stabilizer (SurModics, East Prairie, MN) on a plate shaker at room temperature. After removing of the blocking buffer plates were incubated for 4 hours in a vacuum drying chamber. Dry plates where then stored inside an aluminum pouch with desiccator at 4°C until use. Serum samples were diluted 1∶50 in PBS buffer with 0.3% v/v Tween-20 and 5% w/v instant nonfat dry milk powder. Following 30 minute incubation at room temperature on a plate shaker, the plate was washed 4 times with PBS- Tween. Protein A conjugated with horseradish peroxidase (Southern Biotech, Birmingham, AL) was added to each well, 100 µL of a 1∶2,000 dilution in PBS- Tween, and incubated for 30 minutes at room temperature on a plate shaker. After 4 PBS-Tween washes, 100 µl per microwell peroxidase substrate TMB (SureBlue, 3, 3′, 5, 5′ –Tetramethylbenzidine, KPL, Gaithersburg, MD) was added and plates were incubated for 5 minutes at room temperature on a plate shaker. The peroxidase reaction was stopped by adding 100 µL of 1 M H_2_SO_4_ (Analyzed ACS Reagent, J.T. Baker, Phillipsburg, NJ). Plates were read at A_450 nm_ using a VersaMax® Kinetic ELISA Microplate Reader with SoftMax® Pro v5.4 Software (Molecular Devices Corporation, Palo Alto, CA).

### UCP-rT24H assay

Directly before use, the stock solution of the UCP-ProtA conjugate was suspended by vortex for 10 sec. The desired amount of UCP reporter was diluted in LF assay buffer to a final volume of 100 µL and sonicated (2 cycles of 30 s at 20 kHz and 320 W power with 15 s power off between, in a ultrasonic water bath, Biorupter Diagenode) to homogenize potential aggregates. UCP reporter was further diluted in assay buffer to 7.15 ng/µL for both the 400 nm and 40 nm particles. The UCP-rT24H assay consists of three consecutive flow (CF) steps: 40 µL of diluted sample (2.5% v/v serum sample in assay buffer), followed by a 20 µL wash step (assay buffer), and finally 70 µL UCP-ProtA conjugate (500 ng), following the earlier described protocol for antibody detection [31] with a 2 min pause after flow step 1 (sample) and a 5 min pause after flow step 2 (wash). Chromatography was allowed to continue for at least 30 min or until strips were completely dry. LF strips that are still wet upon scanning may generate a lower emission signal; if needed, LF strips were dried in an oven after a minimum flow time of 30 min. LF strips were scanned by using a multi-strip scanner, modified Fluorocount Packard reader [25], [43] and the a custom ESEQuant *LFR* (QIAGEN Lake Constance, Stockach, Germany) adapted for excitation at 980 nm and emission at 550 nm. The latter reader is a small, lightweight reader suited for single strip scanning; it is also referred to as UCP-Quant [12], [42]. Emission signals measured at the T and FC line were measure in relative fluorescent units (RFU, Packard) or in mV (UCP-Quant); Ratio values (calculated by dividing T by FC) were used to analyze test results rather than using T signals only [43].

### Data analysis

Peak areas for T and FC line were calculated by the reader-specific software. The Packard reader used OTI-Connect (version 1.1.23, OraSure Technologies) and the UCP Quant reader used Lateral Flow Studio (version 3.03.05, QIAGEN Lake Constance). Data were entered into Excel, T line signals were divided by the FC line signals of each individual strip delivering the Ratio (T/FC) value as described previously [43]. Additionally, to express data as a normalized assay value (NAV) relative to the highest quality control sample, T line signals or Ratio values were presented as the relative value (in percentage) compared to the highest value obtained with the 100 Units standard in a particular experiment.

## Results

### UCP-rT24H assay conditions

#### Serum sample load

The performance of the UCP-rT24H assay was first assessed with a set of sera with reactivities ranging from 1 to 100 Units as determined by ELISA. The 2.5 Units sample represents the targeted lower limit of detection (LLOD), a sample with low antibody titer. The general protocol applied for the UCP-LF assay used for the majority of the experiments in this study, implied a dilution of sera in assay buffer such that 1 µL undiluted serum was delivered to the LF strips during the first flow step of the CF protocol (Fig. 1). The assay seemed to perform similarly to previously described UCP-CF antibody assays indicating high degree of flexibility for sample input [31], [32], [46].

#### Amount of rT24H antigen on the test (T-)line

**Figure 2:**
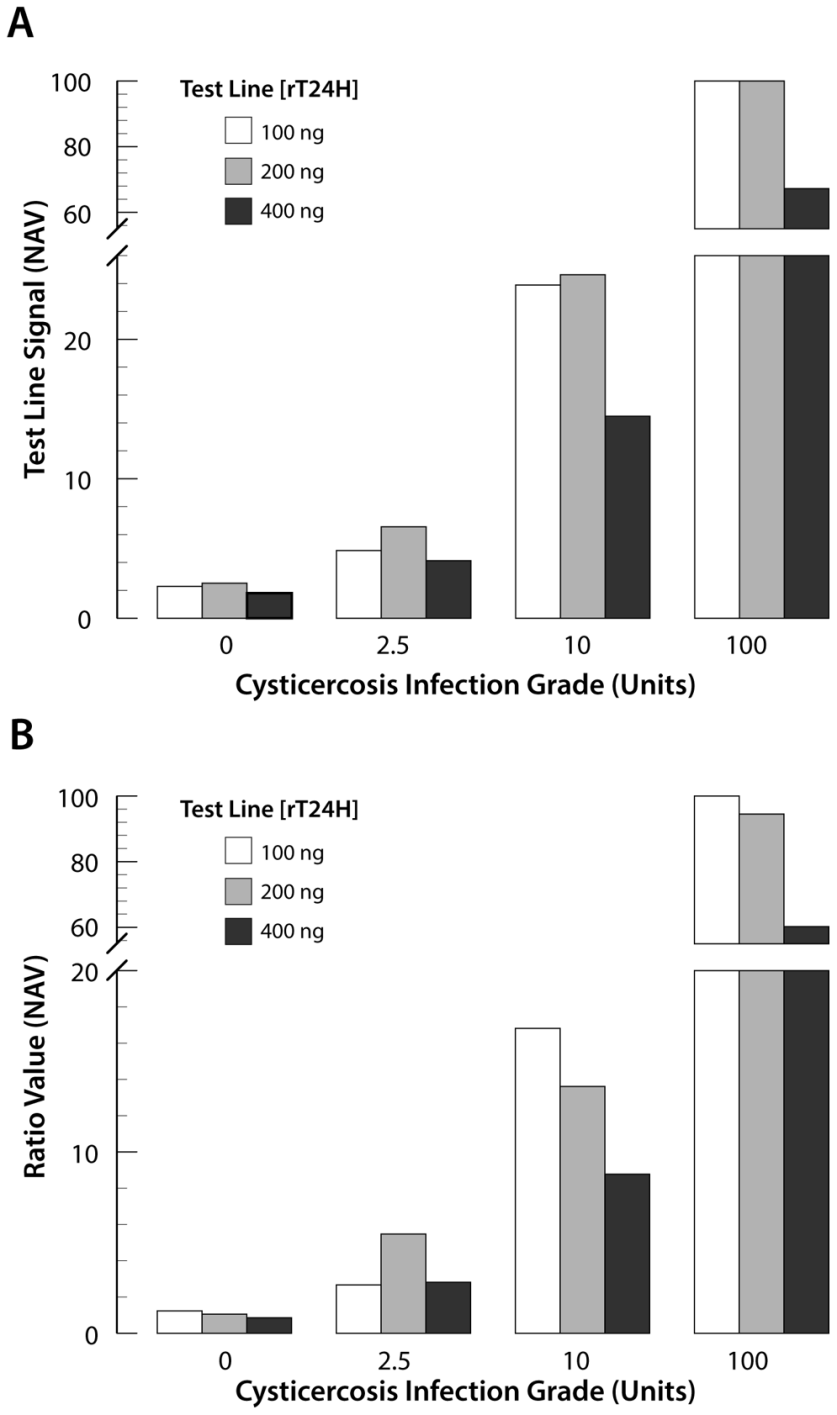
Optimization of rT24H capture antigen load of the T-line. Performance of the UCP-rT24H assay with a standard reference panel of cysticercosis serum samples. The T-line signal (panel A) and Ratio value (panel B) indicate an optimum for the 2.5 Units sample with the 200 ng rT24H Test. Assay results are presented as normalized assay values, as percentage of the highest signal obtained with the 100 Units standard.

Often, the major production cost of LF-based assays is linked to the capture antigen on the T-line. The density (amount) of specific-antigen must be sufficient to capture the target molecules yet not in excess so to lead to poor immobilization of the capture antigen on the LF strip, thereby resulting in the unexpected loss of signal. For the UCP-rT24H assay the T-line is comprised of purified rT24H. Fig. 2A shows the result of a typical experiment, indicating lower T-signal due to release when using 400 ng of rT24H antigen (per 4 mm width). All assays were performed with the same amount of UCP label, and T-signal values were normalized to the highest T-signal measured with the 100 Units sample; achieved with the LF strips containing an rT24H density of 100 ng, the 200 ng strips scored only a slightly lower signals. Differences become more pronounced when looking at normalized Ratio values (T-line signal divided by FC-line signal). An optimum around the targeted lower limit of detection (LLOD) of 2.5 Units with LF strips containing a T-line comprised of 200 ng rT24H seems apparent. A large difference between the zero and the sample indicative for the LLOD is essential to determine a solid assay cutoff threshold. The relative differences in Ratio values determined for the 0 and 2.5 Units samples were a factor of 2.16, 5.20 and 3.32 for the 100, 200 and 400 ng strips, respectively; corresponding A_450_ ELISA values (not shown) indicated a factor of 2.86. These values may differ when using differently sized UCP particles; the experiment shown in Fig. 2 was performed with 400 nm particles, similar results were observed with the 40 nm particles. An additional constraint to consider is the sensitivity of the applied UCP-LF strip scanner, which is the lowest UCP signal that can be measured with a given UCP reader. Further reduction of the amount rT24H (down to 25 ng) decreased T-line signals such that the 2.5 Units standard sample was not detectable with the ESE Quant reader (results not shown).

#### Submicron- versus nano-sized UCP particles

**Figure 3:**
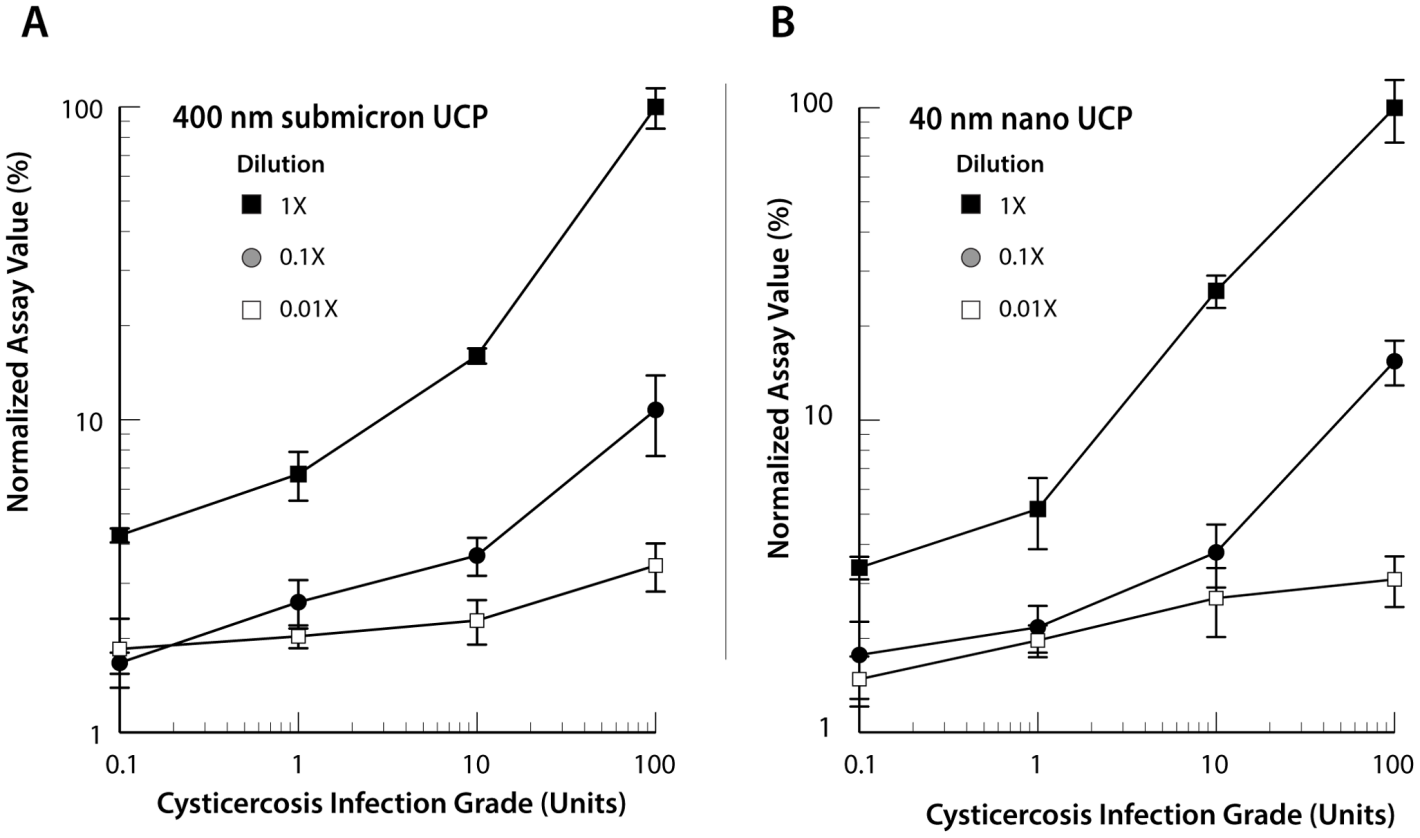
Lower limit of detection with sub-micron and nano-sized UCP particles. Analysis (in triplicate) of standard reference samples (diluted in NHS) with an infection grade of 0, 1, 10 and 100 Units analyzed with the UCP-rT24H assay utilizing submicron-sized 400 nm UCP particles (panel A) and 40 nm nano-sized UCP particles (panel B). Assay results are presented as normalized assay values, as percentage of the highest signal obtained with the 100 Units reference.

The potential of a new source of UCP particles, nano-sized 40 nm particles, was tested on LF strips containing T-lines with 200 ng rT24H antigen. In these experiments the 1 Unit standard sample was included rather than the 2.5 Units standard to allow exploration below the targeted LLOD, set a 2.5 Units. Four standard samples (0, 1, 10 and 100 Units) were diluted 10- and 100-fold in NHS and analyzed with UCP-T24H assay using UCP conjugates made with the 40 nm and 400 nm reporter particles. Fig. 3 shows the result of an experiment performed in triplicate. Assay values were normalized to the highest Ratio value obtained with the 100 Units sample. Results indicate that both particle sizes appear to allow discrimination of the 1 Unit standard. However, the relative difference between the negative control (0 Units) and 1 Unit sample is minimal (a factor of 1.54 and 1.57 for 40 and 400 nm, respectively) and may be difficult to reproduce when experiments are performed as single measurements. Dilution of the original standards in NHS had some effect on the background signal (Fig. 3), indicating that different negative sera will show some variability and as such impact the cutoff threshold of the assay. Overall, the applied test conditions (500 ng UCP-conjugate per strip) seem to be somewhat in favor of the 40 nm nano-particles. This is demonstrated by the increase of the signal strength observed for 1 to 10 Units for the 40 nm particles: a factor of 7.69 versus 3.75 increase for the 40 and 400 nm particles, respectively. The 500 ng UCP particles per LF strip matched well with the lightweight UCP-Quant strip reader available for the analysis. Note that the submicron-sized 400 nm UCP particles applied in other UCP-LF assays [31], [46] were generally used at 100 ng per strip; when comparing equal mass of the 40 and 400 nm UCP particles striped on LF strips, the intensity of emission did not show a relevant difference (results not shown).

### Cutoff threshold and clinical parameters

The above established UCP-LF assay conditions used to validate the UCP-rT24H neurocysticercosis antibody assay involved the use of 4 mm width LF strips with a T-line of 200 ng rT24H and the addition of the equivalent of 1 µL undiluted serum sample and 500 ng UCP protein-A coated reporter particles. Testing of the clinical samples was performed in parallel with using both types of UCP reporter particles: The 40 nm NaYF_4_:Yb^3+^,Er^3+^ particles with poly(acrylic acid) surface and the 400 nm sized Y_2_O_2_S:Yb^3+^,Tm^3+^ particles with a silica coated carboxyl-functionalized surface.

#### Cutoff threshold

In order to assess clinical specificity, the assay cutoff threshold was evaluated with two sets of sera samples from healthy individuals following a protocol as described earlier [47] implying the definition of a low and high specificity cutoff threshold. The UCP-rT24H Ratio values were determined for both sample sets (92 Dutch blood donors and 78 healthy U.S. residents) using both types of UCP particles. Table 1 summarizes the determined values; the low specificity cutoff threshold was defined as the average Ratio value plus two times the standard deviation and the high specificity cutoff threshold was defined as the highest Ratio value in the control group plus two times the standard deviation. Samples generating Ratio values below the low specificity cutoff will be considered antibody negative with the UCP-rTH24 test, samples above the high specificity cutoff will be considered antibody positive. To determine the most likely classification of samples generating signals between the low and high specificity cutoff, the determined threshold values need to be evaluated with a large, statistically relevant, set of confirmed positives. The significant difference in cutoff values when using NaYF or YOS UCP particles is a technical issue that can be regulated by changing assay conditions (e.g. the amount of UCP particles or the amount of rT24H on the Test line). The observed smaller difference in cutoff value between the two sets of healthy individuals tested with the same UCP particles may indicate an effect based on cultural behavior and/or ethnicity.

**Table 1:** Cutoff threshold of the UCP-rT24H assay.

	92 Dutch blood donors		78 U.S. residents	
	40 nm NaYF	400 nm YOS	40 nm NaYF	400 nm YOS
Ratio: Average Value (AV)	0.029	0.008	0.046	0.009
Ratio: Highest Value (HV)	0.110	0.026	0.186	0.033
Standard Deviation (SD)	0.021	0.005	0.032	0.007
Low specificity cutoff: AV+2SD	0.070	0.019	0.109	0.023
High specificity cutoff: HV+2SD	0.151	0.037	0.231	0.047

#### Single blinded assay validation

**Figure 4:**
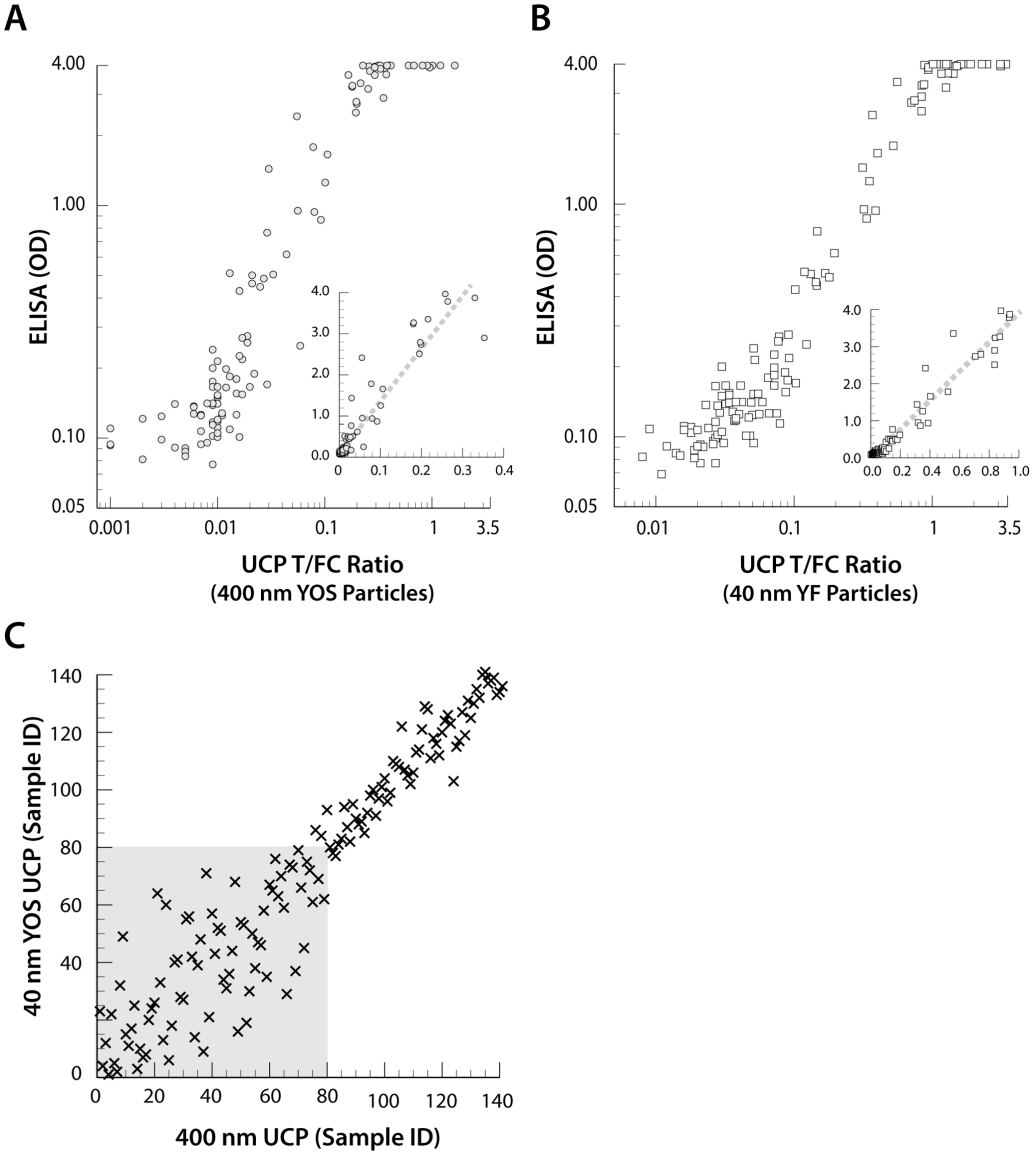
Comparison of the UCP-rT24H assay with the ELISA. Single blind evaluation with 141 clinical samples. Comparison of Ratio values obtained with the 40(panel A) and the 400 nm sized YOS UCP particles (panel B) with the ELISA OD_450_ values. Note that both insets show the results using a linear scale and the *x*-axis limited to 0.4 and 1.0, respectively. Spearman ranking (panel C) of the UCP-rT24H Ratio values obtained with both UCP particles. The grey box indicates samples scoring values below the low specificity threshold (U.S. resident control group).

Validation of the assay was performed using the sera from 63 cases of neurocysticercosis randomly arranged between the set of 78 serum samples from healthy U.S. residents. The resulting 141 samples were tested with both types of UCP particles in a single blind experiment. Obtained UCP-rT24H Ratio values were plotted against the corresponding rT24H ELISA OD_450_ values (Fig. 4) showing a good correlation between the UCP and ELISA. The test conditions for the ELISA were set for best resolution in the low reactive range, implying a maximum A_450 nm_ value of 4 and thus no discrimination between highly reactive samples. As observed when testing the cutoff threshold samples, the Ratio values determined with the 40 nm NaYF UCP particles on average differed by a factor of ∼4 compared to the Ratio values determined with the 400 nm YOS UCP particles. Qualitatively both type of UCP particles seem to perform quite similarly, whereas quantitatively the 40 nm NaYF UCP particles seem to correlate somewhat better with the ELISA. Fig. 4C, a scatter plot of the results obtained with the two types of UCP particles, shows the Spearman correlation (*R* ^2^ is 0.90) of the Ratio values by rank order. The relatively large scattering of points in the lower range is directly linked to the low Ratio values measured for the non-reactive samples (indicated by the grey box).

#### Clinical sensitivity and specificity

Application of the low and high specificity thresholds (Tab. 1) as determined with the 170 healthy controls indicated a number of UCP-rT24H false positives and false negatives. A sample was classified false positive (FP) when it was part of the set of healthy controls with a Ratio value score above the cutoff threshold; a sample was classified as a false negative (FN) when it was part of the set of defined cysticercosis set (with 2 or more cysts identified by imaging). When using both low and high specificity thresholds, an indecisive (IND) or potentially positive group can be identified from samples scoring Ratio values between the low and high specificity threshold, Table 2. By definition, at 100% clinical specificity (Sp) all 170 healthy control samples should score a Ratio value below the cutoff threshold. For the assay with the 40 nm NaYF UCP particles, for example, this is achieved when using the high specificity threshold determined for the U.S. resident group. Clinical sensitivity (Sn) of the assay then drops to 85%; for the 400 nm YOS UCP particles at 100% specificity, the sensitivity is 89%. The highest sensitivity, obtained when applying the low specificity threshold determined for the Dutch blood bank donors, is 98% with a specificity of 88%. The actual required cutoff threshold is depending of the clinical sensitivity and specificity required demanded for this assay; in this respect the area of receiver operating characteristic (ROC) curves of the UCP-rT24H indicate an area of 0.99 for both types of UCP particles. In this particular test the low specificity threshold determined for the U.S. resident group seems to deliver acceptable sensitivity/specificity levels of 96%/98% (Youden's index, J = 0.933) and 94%/98% (J = 0.919) for the 40 and 400 nm UCP particles, respectively. For both particles, these numbers imply a positive and negative predictive value of 94% and 98%, respectively. With the ELISA 96% sensitivity is achieved with 94% specificity (J = 0.898). These numbers indicate at least equivalent performance of the UCP-rT24H assay as compared to the ELISA. Moreover, results show that in consecutive flow based assays [31] the 40 nm NaYF UCP are a good alternative for the 400 nm YOS UCP particles.

**Table 2:** Performance of the UCP-rT24H assay.

UCP Reporter	40 nm NaYF UCP particles				400 nm YOS UCP particles			
Control group	Dutch donors		U.S. residents		Dutch donors		U.S. residents	
**Specificity threshold**	**Low**	**High**	**Low**	**High**	**Low**	**High**	**Low**	**High**
Indecisive (IND)	0	8	0	10	0	7	0	8
***False negative (FN), IND included***	1	1	3	1	2	4	4	0
FN without IND	1	9	3	11	2	11	4	8
False positive (FP) in Dutch group	6	0	1	0	5	0	2	0
FP in U.S. group	18	1	3	0	7	0	2	0
***FP, Dutch+U.S. group***	24	1	4	0	12	0	4	0
Clinical Sensitivity (Sn)^a^	98.4%	87.5%	**95.5%**	85.1%	96.9%	85.1%	**94.0%**	88.7%
Clinical Specificity (Sp)^b^	87.6%	99.4%	**97.7%**	100.0%	93.4%	100.0%	**97.7%**	100.0%
Youden's index, J	0.863	0.870	**0.933**	0.851	0.905	0.851	**0.919**	0.887

a Sn was calculated dividing the number of true positives (TP) by the sum of the number of TP+FN; TP are the 63 classified cysticercosis samples (Peru sample set).

b Sp was calculated dividing the number of true negatives (TN) by the sum of the number of TN+FP; TN are the 170 healthy control samples (Dutch and U.S. sample set).

#### Heterologous infections

A challenging set of serum samples from individuals with other parasitic infections was tested with the 40 nm NaYF UCP particles to evaluate cutoff threshold and specificity. The set of 80 samples amongst others included serum samples from individuals infected with *Hymenolepis nana* (dwarf tape worm, n = 8), *Trichinella spiralis* or *Wuchereria bancrofti* (round worms, n = 15), long-, liver-, or intestinal-flukes (n = 11), *Schistosoma* species (blood-flukes, n = 20) and samples from individuals carrying mixed (worm) infections. Stool samples did not contain *T. solium* eggs or proglottids. Two *Schistosoma mansoni* samples and one *T. spiralis* sample were reactive with a Ratio signal equivalent to the 20 U standard. One *H. nana* and one *P. westermani* showed a low response, with a Ratio value between the 2.5 and 5 U standard. Assuming that the five reactive samples are false positives (note that involved individuals have not been examined for neurocysticercosis incidents), clinical specificity in this group of 80 is 94.1%. When including the Dutch donor and U.S. residents control samples and using the low specificity cutoff Ratio value as determined with the 40 nm NaYF UCP particles for the 78 U.S. residents (Table 1), clinical specificity in the combined negative group (n = 250) is 96.5%; TN = 250, FP = 4+5.

### Performance of the lightweight portable LF strip reader

**Figure 5:**
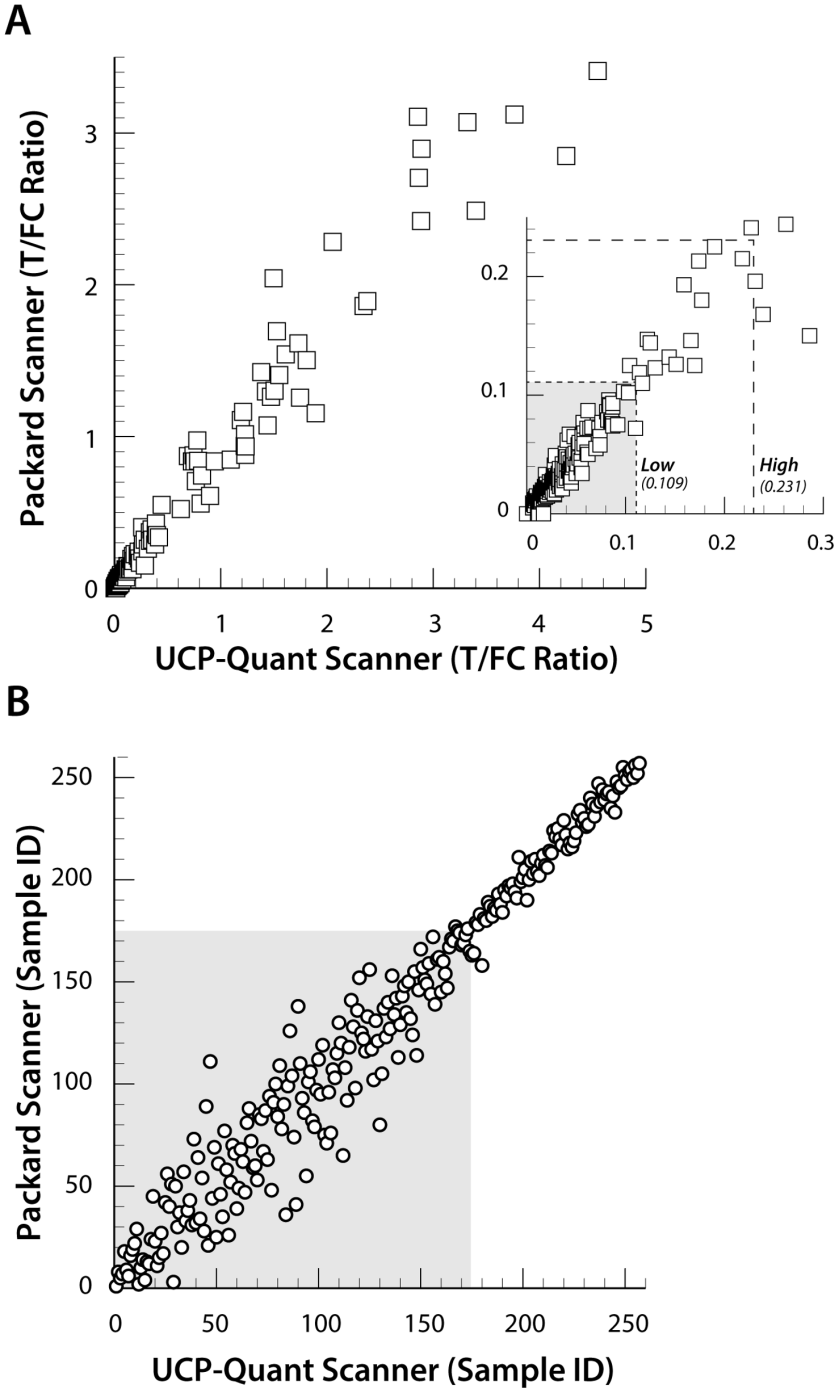
Comparison of the UCP Quant with the Packard reader. Panel A: A scatter plot of UCP-rT24H Ratio values obtained from 257 LF strips calculated. Specificity cutoff thresholds calculated for the U.S. residents control group (Table 1) are indicated. Panel B: A scatter plot showing the samples ranked on UCP-rT24H Ratio value (Spearman rank correlation). The grey box indicates samples scoring values below the low specificity threshold (U.S. residents).

The use of a commercially available LF strip reader platform (ESEQuant *LFR*) custom adapted with UCP capability [12], [32], [42] was evaluated for its use in applications with nano-sized (40 nm) NaYF UCP reporter particles. A total of 258 LF strips obtained from testing clinical samples and various standards series with 40 nm UCP particles were re-analyzed on the adapted ESEQuant *LFR* reader referred to as UCP Quant. Fig. 5 shows the correlation of the results obtained with the lightweight UCP Quant reader and the multi-strip Packard reader. Ratio values are used rather than actual peak areas of the T and FC lines as the determined ratio amongst others can manage device related variations, e.g. difference caused by variation in the performance of different IR light-emitting diode. This allowed direct inter assay comparison of ratio value results when the same type of reader and software is used. In panel A, the Ratio value determined with both readers indicates excellent agreement with a linear correlation coefficient *R* ^2^ = 0.96. Focusing on the lower Ratio value range indicates a cluster of assays results below the cutoff threshold determined for U.S. residents using the multi-strip Packard reader, 0.109 (Tab. 1). This cluster includes most healthy control samples as also indicated in panel B showing the excellent Spearman correlation (*R* ^2^ = 0.96) by Ratio value rank order (low specificity area indicated in grey). The UCP-Quant reader results would not change the outcome of the single blinded clinical validation experiment and thus is an appropriate reader in combination the 40 nm NaYF in the here described CF assay format with 500 ng UCP particles per test. Moreover, even though different software is used to calculate T and FC peak areas and although the Packard reader is equipped with a photo multiplier and a higher power IR laser, the actual ratio values measured with both readers differed only slightly.

## Discussion

This study describes the development of a new low cost screening tool to simplify the diagnosis of neurocysticercosis on-site and at POC facilities, a helpful tool in control programs to estimate sero-prevalence. The serological test builds on a previously described method using LF and the unique UCP reporter label. Evaluation of the new UCP-rT24H test with a set of clinical samples indicated excellent clinical parameters comparable to ELISA. Application of new nano-sized UCP reporter label and a lightweight strip analyzer, both from easily accessible sources, demonstrated equal performance to previously used ‘research and development’-only materials.

The applied UCP-LF format (referred to as consecutive flow, CF) was developed previously to allow simultaneous (multiplex) detection of antibodies against different infectious diseases [31]. This was achieved using LF strips with multiple distinct disease/pathogen specific capture lines and the sequential flow of a clinical sample, followed by assay buffer and a flow of an Ig-specific UCP conjugate. Separation of the sample flow from the UCP-ProtA reporter flow allows the anti-rT24H specific antibodies to bind and enrich at the T line while other human immunoglobulins (Ig's) will flow past the T-line towards the FC line and absorbent pad, before interaction with the UCP-ProtA reporter. As a result, anti-rT24H specific antibodies do not need to compete with the surplus of other human Ig's for binding to the relatively limited number of protein A molecules on the UCP reporter. Detection of antibodies indicating neurocysticercosis is another example showing the applicability of UCP-CF assay format for antibody detection. The described UCP-rT24H assay demonstrated excellent feasibility for detecting neurocysticercosis cases.

A notable difference of the new test is the use of bacterial expressed recombinant antigen rT24H. Currently, the availability of rT24H cysticercosis antigen is limited as a consequence of the relatively complex production method requiring a baculovirus expression system. The initial results shown here appear to validate the use of the bacterial rT24H recombinant antigen, but this will require further analysis. Moreover, further optimization is needed to reproducibly identify sera from patients with single cyst infections. Important also are specificity issues; do LF strips with the bacterial produced rT24H perform similar to LF strips with a capture line of baculovirus produced rT24H? In this study, U.S residents and Dutch blood donors were used as control group for confirmed neurocysticercosis cases from Peru because a defined control group from the endemic area was not available. It is obvious that the use of a non-endemic control group to determine assay cutoff values is not optimal and this may require further validation. However, the performance of the assay with a set of samples with heterologous worm infections demonstrated clinical specificity values in the same range as determined for the two non-endemic healthy control groups. A further evaluation of the current assay should also include appropriate (confirmed neurocysticercosis negative) endemic controls as well as taeniasis cases. The performance of the assay with the current set of clinical samples was comparable to the more established methods EITB-LLGP and the in-house ELISA. Moreover, results obtained with the UCP-rT24H assay do not appear to differ from the previously described ELISA alternative, the MICT assay utilizing baculovirus rT24H [11]. The MICT test however does not include a portable reader to allow analysis of the assay remote from the laboratory.

With this study we have shown proof-of-principle for use of (potential) commercially available UCP reporter materials and analyzer equipment. The production process (thermal composition of sodium and lanthanide trifluoroacetates) of the new 40 nm NaYF particles is well described and easier to control [36]. Brightness of UCP particles (visible emission upon IR excitation) is directly related to the volume of the particle; however, per mass equivalent, the current 40 nm NaYF particles emit approximately the same amount of light (upon IR excitation) as the 400 nm YOS particles. In general, smaller particles will need fewer capture molecules to bind to the Test line of a LF strip. Whether the current size and shape is optimal can be determined empirically as the UCP production process allows a well-controlled particle size and morphology [36]. Additionally, coating of the current 40 nm particles with an inert silica layer instead of the current poly (acrylic acid) polymer surface may further improve UCP particle performance in LF-based assays. A robust coating is also of importance when moving towards dry assay reagents [12].

The two different control groups of healthy individuals used in this study to validate the assay suggested that differences in cultural behavior and/or ethnicity may affect analytical sensitivity (the lower limit of detection). However, as these difference between Europeans and U.S. residents probably are minimal, the observed difference may also be a consequence of using banked serum samples that were collected, pre-treated (serum preparation) and stored at different locations. Also, in order to make this test conveniently suitable for on-site use or point-of-care testing the assay should allow the use of fresh whole blood finger stick samples. The use of finger-stick blood in UCP-LF based assays is part of ongoing research for the quantitative testing of trough levels of immunotherapeutics [32]. Antibody testing using saliva as a non-invasive specimen type is also an attractive area for exploration when aiming at field testing, but this will require a comprehensive reanalysis of the cutoff [15].

In conclusion: We have demonstrated that an earlier described rapid UCP-CF serological assay format can be applied to detect antibodies produced during neurocysticercosis. The assay performed well with LF strips containing an antibody-capture line comprised of bacterial expressed recombinant T24H, a convenient alternative for current rT24H produced with a baculovirus expression system. The new 40 nm NaYF UCP particles and the custom adapted lightweight semi hand-held LF strip reader are appropriate alternatives for earlier used and difficult to access reporter material and more expensive laboratory-based scanning equipment.

## Supporting Information

Figure S1 **Purity of the bacterial T24H fusion protein.** The bacterial expressed T24H used in the current UCP-LF assay is a GST-T24H fusion protein with a molecular weight of 34 kDa. To assure production of sufficient full-length T24H, it was expressed as a double-tagged protein, with a His tag at the C-terminus and the GST tag at the N-terminus. After a two-step purification protocol involving both tags, the His-tag was removed but the GST-tag was retained. The GST-tag (24 kDa) allowed convenient imagining of the GST-T24H fusion protein on a Coomassie Brilliant Blue (CBB) stained denaturing gel (SDS-PAGE); the 10 kDa T24H by itself does not stain well due to a low percentage of aromatic amino acids. The GST tag does not interfere with the specificity of the T24H assay and improved binding of the antigen to the LF strip. Purity of the GST-T24H fusion protein (F, 34 kDa) was analyzed using CBB-stained SDS-PAGE. The major part of the purified material is full length GST-T24H 34 kDa fusion protein (*F*, lane 2); the less abundant smaller bands indicate some degradation of the C-terminus of the T24H. The presence of the 10 kDa T24H (*T*) fragment was demonstrated after thrombin cleavage of the 24 kDa GST tag (*G*); left and right lanes (lanes 1 and 4) contain molecular weight (MW) markers. After thrombin cleavage a single 24 kDa band is evident (*G*, lane 3), the T24H fragment (*T*, lane 3) is visible as a less distinct and poorly stained band.
(TIF)

Figure S2 **ELISA comparison of bacterial-expressed rT24H with reference antigen.** To validate the performance of the bacterial expressed T24H (GST-T24H fusion protein), a set of 39 serum samples (including 13 confirmed cysticercosis and taeniasis cases) were analyzed by ELISA utilizing both the new bacterial-expressed rT24H and the reference antigen, rT24H produced using the baculovirus production system. In panel A, the plot of ELISA A_450_ values of both rT24H preparations. Panel B shows the performance around the theoretical cutoff thresholds (indicated by the dotted lines, experiment performed in singlet). In panel C the Spearman correlation with samples ranked by order of their A_450_ value; indicated cutoff thresholds (dotted lines) correlate with the thresholds shown in panel B. Based on determined assay value (ELISA), results demonstrate a high correlation indicating that both antigens performed similarly for this set of samples.
(DOCX)
