# Supplementary figures and images for: Feasibility of a Lateral Flow Test for Neurocysticercosis Using Novel Up-Converting Nanomaterials and a Lightweight Strip Analyzer
Source: PLoS Negl Trop Dis. 2014 Jul 3;8(7):e2944. doi: 10.1371/journal.pntd.0002944 (PMC4080996; doi:10.1371/journal.pntd.0002944)

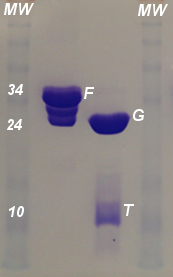

Supplement: Figure S1 — Purity of the bacterial T24H fusion protein. The bacterial expressed T24H used in the current UCP-LF assay is a GST-T24H fusion protein with a molecular weight of 34 kDa. To assure production of sufficient full-length T24H, it was expressed as a double-tagged protein, with a His tag at the C-terminus and the GST tag at the N-terminus. After a two-step purification protocol involving both tags, the His-tag was removed but the GST-tag was retained. The GST-tag (24 kDa) allowed convenient imagining of the GST-T24H fusion protein on a Coomassie Brilliant Blue (CBB) stained denaturing gel (SDS-PAGE); the 10 kDa T24H by itself does not stain well due to a low percentage of aromatic amino acids. The GST tag does not interfere with the specificity of the T24H assay and improved binding of the antigen to the LF strip. Purity of the GST-T24H fusion protein (F, 34 kDa) was analyzed using CBB-stained SDS-PAGE. The major part of the purified material is full length GST-T24H 34 kDa fusion protein (F, lane 2); the less abundant smaller bands indicate some degradation of the C-terminus of the T24H. The presence of the 10 kDa T24H (T) fragment was demonstrated after thrombin cleavage of the 24 kDa GST tag (G); left and right lanes (lanes 1 and 4) contain molecular weight (MW) markers. After thrombin cleavage a single 24 kDa band is evident (G, lane 3), the T24H fragment (T, lane 3) is visible as a less distinct and poorly stained band. (TIF) [file pntd.0002944.s001.tif]
