# Supplementary material for: Feasibility of a Lateral Flow Test for Neurocysticercosis Using Novel Up-Converting Nanomaterials and a Lightweight Strip Analyzer
Source: PLoS Negl Trop Dis. 2014 Jul 3;8(7):e2944. doi: 10.1371/journal.pntd.0002944 (PMC4080996; doi:10.1371/journal.pntd.0002944)
Supplement: Supplementary file 2 [file pntd.0002944.s002.docx]

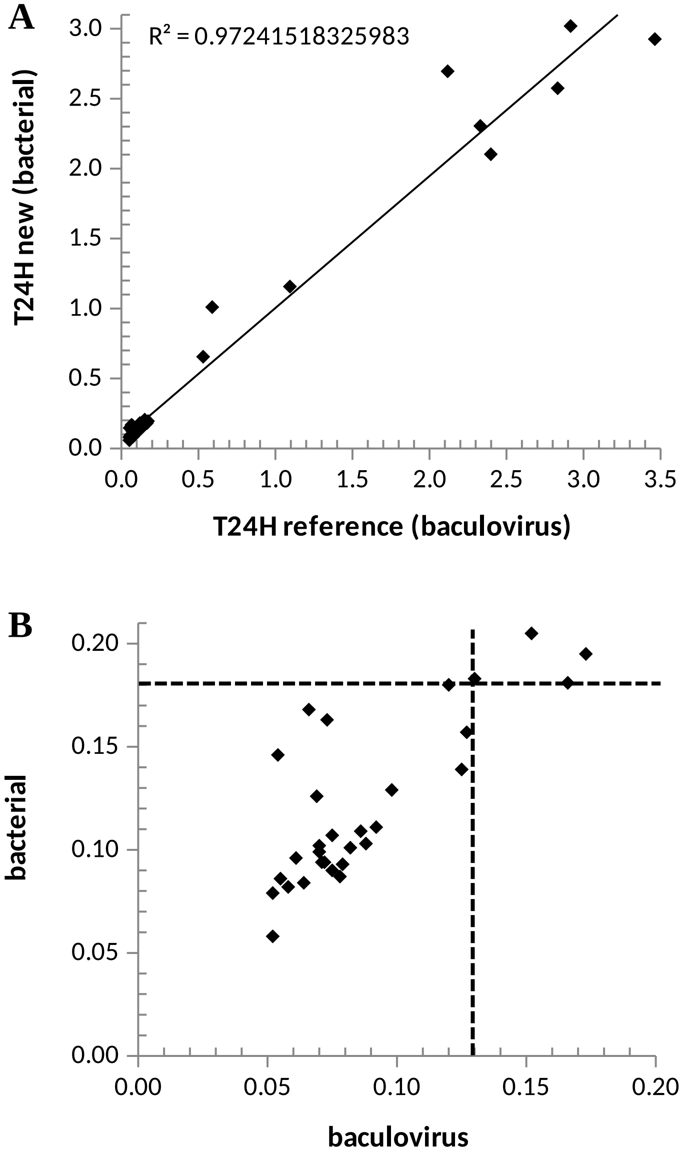
A
R² = 0.97241518325983
T24H new (bacterial)
T24H reference (baculovirus)
0.0
0.5
1.0
1.5
2.0
2.5
3.0
3.5
B
bacterial
baculovirus
0.00
0.05
0.10
0.15
0.20

C

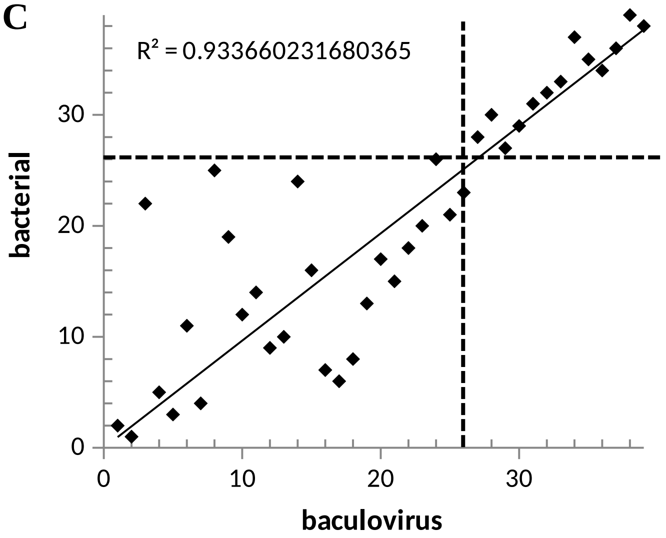

R^2 = 0.933660231680365
30
20
10
0
0
10
20
30
bacterial
baculovirus
